# MetaGeniE: Characterizing Human Clinical Samples Using Deep Metagenomic Sequencing

**DOI:** 10.1371/journal.pone.0110915

**Published:** 2014-11-03

**Authors:** Arun Rawat, David M. Engelthaler, Elizabeth M. Driebe, Paul Keim, Jeffrey T. Foster

**Affiliations:** 1 Pathogen Genomics Division, Translational Genomics Research Institute, Flagstaff, Arizona, United States of America; 2 Center for Microbial Genetics and Genomics, Northern Arizona University, Flagstaff, Arizona, United States of America; 3 Department of Molecular, Cellular, and Biomedical Sciences, University of New Hampshire, Durham, New Hampshire, United States of America; University of British Columbia, Canada

## Abstract

With the decreasing cost of next-generation sequencing, deep sequencing of clinical samples provides unique opportunities to understand host-associated microbial communities. Among the primary challenges of clinical metagenomic sequencing is the rapid filtering of human reads to survey for pathogens with high specificity and sensitivity. Metagenomes are inherently variable due to different microbes in the samples and their relative abundance, the size and architecture of genomes, and factors such as target DNA amounts in tissue samples (i.e. human DNA versus pathogen DNA concentration). This variation in metagenomes typically manifests in sequencing datasets as low pathogen abundance, a high number of host reads, and the presence of close relatives and complex microbial communities. In addition to these challenges posed by the composition of metagenomes, high numbers of reads generated from high-throughput deep sequencing pose immense computational challenges. Accurate identification of pathogens is confounded by individual reads mapping to multiple different reference genomes due to gene similarity in different taxa present in the community or close relatives in the reference database. Available global and local sequence aligners also vary in sensitivity, specificity, and speed of detection. The efficiency of detection of pathogens in clinical samples is largely dependent on the desired taxonomic resolution of the organisms. We have developed an efficient strategy that identifies “all against all” relationships between sequencing reads and reference genomes. Our approach allows for scaling to large reference databases and then genome reconstruction by aggregating global and local alignments, thus allowing genetic characterization of pathogens at higher taxonomic resolution. These results were consistent with strain level SNP genotyping and bacterial identification from laboratory culture.

## Introduction

Despite its importance for infectious disease diagnosis, the ability to rapidly and conclusively identify the causative agents for infections remains an elusive goal. When a symptomatic patient enters the healthcare system, the infectious etiologic agent is rarely known. Patients often are subjected to a battery of expensive tests, often taking days to weeks for results, to narrow down the etiological agent; meanwhile the treating physician is typically forced to make management decisions based on patient symptomology and history. Next-Generation sequencing technologies have transformed our ability to rapidly generate sequence data [1]–[3]; and as such, whole metagenome sequencing is emerging as the future of clinical diagnostics by providing a rapid and highly sensitive method of diagnosing and characterizing infectious agents in clinical samples [4]–[9]. The goal is to replace the multitude of clinical microbiological tests with a single diagnostic approach. In clinical metagenomic analysis, microbial and host DNA are sequenced together and the likely pathogens identified and characterized to streamline treatment. Despite this seemingly simple process, there are numerous obstacles to efficient and accurate identification of pathogens in clinical samples.

Over the past 5–10 years, the composition of microbial communities (i.e., the microbiome) in clinical samples, and elsewhere, has been estimated using conserved gene amplicon sequencing (e.g., 16S rRNA for bacteria). More recently, whole genome sequencing (WGS) approaches have emerged as a powerful alternative that gives a relatively unbiased and global representation of the members of the microbial community [7], [10], [11]. With the advances in sequencing technology, along with decreasing cost, it is now possible to fully interrogate the microbial communities within clinical samples [6], [12], [13], including the ability to genotype community members and understand gene composition. This diagnostic advancement can provide important insights for accurate and timely clinical management of patients. For clinical diagnostics, genus- or even species-level identification may not be sufficient for proper clinical treatment. For example, a patient suffering from methicillin resistant *Staphylococcus aureus* TCH1516 requires different treatment than a patient colonized by methicillin sensitive *S. aureus* Newman.

A primary issue for metagenomic analyses is read alignment methodology, for analysis of the hundreds of millions of reads per run generated through sequencing technologies [14], [15]. Different metagenomic analysis pipelines incorporate available aligners (local/global) in a computational infrastructure, such as cloud computing or high performance computing (HPC), to provide accurate sequence interrogation, computational speed and the scalability necessary to query enormous numbers of metagenome reads against reference databases. There is, however, a tradeoff between the accuracy of detection and computational speed. Local alignment algorithms are considered to be more sensitive and accurate than global alignment algorithms [16], [17]. On the other hand, existing global aligners [18]–[26] are typically preferred over local aligners, given the high volumes of metagenome sequences [27]. For example, PathSeq [6], MePIC [28] and SURPI [29] utilize cloud computing platforms to expand computational scalability. These computing platforms are usually available externally or commercially and have associated utilization costs but do not require server maintenance costs by the user. PathSeq, IMSA [30], VirusHunter [31] and MEGAN [32] are capable of characterizing unknown reads with BLAST, a local aligner [33]; however, with the high number of metagenome reads (>1 million), BLAST is often not optimal for clinical diagnostics given the high computational time required [16], [17]. RINS [34] and IMSA [30] invoke processes such as BLAT [35] but without parallelization and consequently have scalability issues with large reference databases.

An additional issue beyond alignment methodology is read assignment. Each metagenomic sequencing read, in theory, originates from a single genome. Assigning large numbers of reads (especially 50–200 bp short reads) back to their genome of origin is problematic for multiple reasons including: a) the presence of overlapping/shared genomes from other organisms in the sample; b) querying these reads against related genomes from publicly available databases may result in a greater number of hits due to homology; and c) the computational resources required to scan through large reference databases. We have developed a pipeline, MetaGeniE, which has been designed for accurate, sensitive and specific detection of taxa in complex microbial samples and to address all of the above limitations with typical metagenomic analyses. The MetaGeniE pipeline generates an all-against-all comparison dataset between the reads and the reference database and then uses these results to generate cumulative statistics from combined local and global alignment. MetaGeniE also incorporates features such as comprehensive human read filtration and scalability to search large reference databases such as the microbial Refseq database (http://www.ncbi.nlm.nih.gov/refseq/), which is increasing with each release and presently around 20 GB in size.

## Methods

### Ethics Statement

All work with tissues derived from human subjects was approved by the Institutional Review Boards of Northern Arizona University and the Translational Genomics Research Institute. Both Institutional Review Boards waived the need for patient consent for these de-identified samples.

### Data

#### Human Datasets

Seven whole genome sequences of human datasets were downloaded from Sequence Read Archive (SRA) at NCBI (http://www.ncbi.nlm.nih.gov/sra/). The accessions and read number for these datasets are ERR191896: 53.03 million reads; ERR218094: 49.50 million reads; ERR237515: 2.54 million reads; SRR032752: 35.29 million reads; SRR033605: 23.53 million reads; SRR054743: 40.63 million reads; SRR054753: 39.76 million reads. We simulated 30 million reads from human reference genome (build 37.2) (ftp://ftp.ncbi.nih.gov/genomes/H_sapiens) with GRINDER version 0.5.3 [36]. We incorporated total 0.5% variability in the simulated human reads, 0.1% as expected human SNP frequency [19] and 0.4% as the average sequencing error for Illumina reads [37].

#### Bacterial Datasets

Average Illumina sequencing error of 0.4% was incorporated in all the simulated reads generated from bacterial reference genomes (ftp://ftp.ncbi.nih.gov/refseq/release/bacteria/). To study sequencing error and its effect on detection and characterization, additional variability of 0.1%, 0.2%, 0.5% and 1% were incorporated in each simulated bacterial library.

#### In-house Clinical Dataset

Three throat swabs (CF1, CF2, CF3) and one nasopharyngeal swab (CF4) from cystic fibrosis (CF) patients were sequenced with Illumina GA IIx using paired-end 100 bp reads (total reads ∼37–58 million). Culture-based methods were also performed for the CF samples to identify microbial infection.

We benchmarked our work using only simulated Illumina reads since this is currently the leading sequencing platform in overall usage and its high throughput provides an opportunity to test computational scalability. The pipeline can utilize other platforms and as expected, the detection will incorporate platform-specific biases [38].

### Design

**Figure 1:**
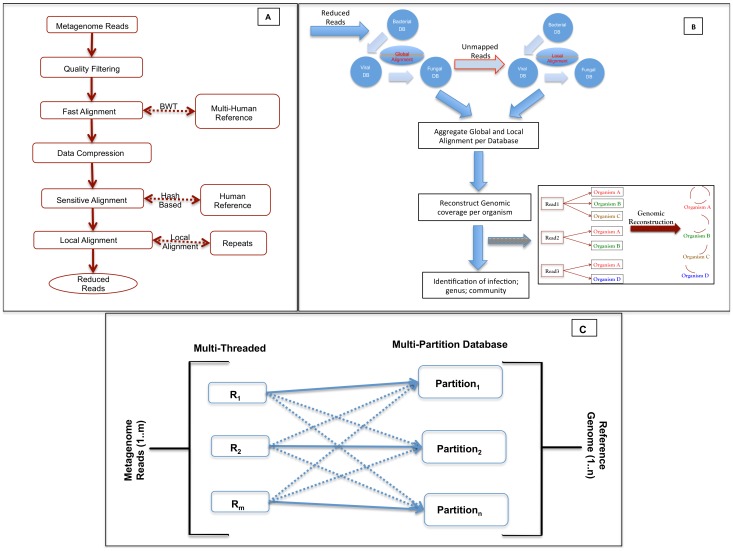
The workflow of the pipeline. **A.** Human read reduction module **B.** Pathogen detection module **C.** Multithreaded input sequence file query the multiple partition reference database to address the scalability.

The pipeline is designed as a distributed and scalable software package to analyze millions of reads and query large reference databases and consists of two modules: Read-Reduct and Patho-Detect. The Read-Reduct module sequentially filters and reduces the low quality, redundant, and human reads (Figure 1-A). The low quality reads are filtered using PRINSEQ [27]. Human read filtration can be performed with the short read aligners that are classified into Burrows-Wheeler Transform (BWT) mappers and hash-based mappers. The BWT mappers such as BWA, SOAP2 and Bowtie are fast but considered less sensitive, while the hash-based aligners are slow but more accurate such as MAQ, ELAND, Novoalign and STAMPY [19]. To reduce overall computational processing time and memory, one of two faster BWT aligners, BWA [18] or BOWTIE2 [21], are utilized initially in the pipeline. Higher CPU and memory intensive features such as data compression [27] and hash-based sensitive alignment STAMPY [19] are then utilized to further reduce the overall number of reads. The second module of the pipeline, Patho-Detect, aligns the remaining reads against known bacterial, fungal and viral sequences with BWT alignment followed with the local aligner BLAT [35] (Figure 1-B).

### Scalability

Incorporating a large reference database such as RefSeq rather than using just a few selected complete genomes allows identification to subspecies/strain level for a broad range of taxa. The RefSeq bacterial database has doubled from 8.7 G in Release 54 to 19 G in Release 60 for bacteria and will be increasing in the future. This results in increasing demand for computational memory to scale to sizeable reference databases. To address the issue of scalability with large reference databases, we designed the pipeline to handle multiple partitions of a reference database for better memory management (Figure 1-C). Multithreaded input files query each smaller database partition (∼1 GB) iteratively and thus reduce the overall memory footprint. This querying of each input file fragment generates higher number of mapped-unmapped relationships against the partitioned database results per iteration, which increases the computational time. To address this issue, the pipeline utilizes custom hash functions and indexing tools formatdb and fastacmd (ftp://ftp.ncbi.nlm.nih.gov/blast/executables/) to allow faster extraction of millions of reads as an input for the next reference database search.

### Normalized Genome Coverage

Assessing the detection of a pathogen by the total number of reads that hit/align to the respective genome(s) is not always an accurate predictor of presence of an organism due to repeat elements, close relatives in the metagenome and PCR amplification biases. To overcome these issues, MetaGeniE detects microbial presence by genomic reconstruction, which is the percent of the genome mapped to the reference genome(s) for each organism. The pipeline first converts the local and global alignment output to common BED format. Genome coverage of each mapped organism is then calculated from the global and local alignments with BEDTOOLS [39]. The total genome reconstructed for each mapped organism is the sum of genome coverage from global and local alignments for any metagenome. The normalized genome coverage (% genome coverage) is calculated as follows:
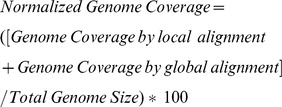



The normalized genome coverage allows comparison of different organisms with different genome sizes, which is helpful in representing the abundance of various organisms in each metagenome for community analysis (See Clinical Samples Section below).

### Computing Infrastructure

To benchmark the performance of the pipeline, all the human datasets and simulated (human and bacterial) datasets were run on the same High Performance Computing (HPC). A 47 GB RAM and 6 processor limit was set for all the simulated and downloaded human datasets. Analysis of the CF clinical dataset was executed with eight processors and 100 GB RAM in a HPC cluster. The CPU hour logs are generated by the pipeline for comparison of runtime between different processes. Simulated datasets were generated in FASTA format without quality values. Downloaded human datasets were filtered at a quality threshold of Phred value >15. Only the quality-filtered sequences were utilized to benchmark, as some samples had higher percentages of low quality reads.

### Benchmarking

To test the sensitivity of the pipeline, synthetic reads 100 bases in length were generated from respective reference genomes with a range of read numbers. These are represented as follows: 0.1K: 100 reads; 1K: 1,000 reads; 10K: 10,000 reads; 100K: 100,000 reads; 250K: 250,000 reads. Human filtration is a five step process and different steps are utilized to compare the sensitivity/specificity of detection of simulated reads, execution speed and memory usage (Table 1). All these steps utilized the same parameters as follows, BWA (default), BOWTIE2 (default with very sensitive mode), STAMPY (default), PHRED quality score >15, minimum length >50, low complexity (dust) and BLAT (80% identity).

**Table 1:** Description of different steps of human filtration of pipeline utilized to compare sensitivity/specificity of detection and performance of runtime and computational resources of the simulated reads.

	Quality Filter	Fast Alignment	Data Compression	Sensitive Alignment	Repeat DB
mg_bw2	yes	bowtie2	-	-	-
mg_bwa	yes	bwa	-	-	-
mg_dc	yes	bwa	-	stampy	yes
mgall_bw2	yes	bowtie2	Yes	stampy	yes
mgall_bwa	yes	bwa	Yes	stampy	yes

Dash (-) represents that the option was not utilized.

### SNP Genotyping

The reads mapping to organisms with the highest genome coverage, as detected by the pipeline, were extracted. Besides genome coverage that is proportional to pathogen DNA (and usually incomplete), other factors like depth, recombinant genomes are factors to be considered for performing SNP genotyping. FASTA formatted sequence files generated from mapped reads can then be used for SNP genotyping for such goals as the identification of specific lineages, fine-scale strain differentiation, and determination of antibiotic resistance variants. We use an in-house SNP Pipeline that integrates the SNPs detected by SolSNP (http://sourceforge.net/projects/solsnp/) from BWA alignment and Mummer 3.22 [40] from available public genomes although other SNP pipelines can be incorporated. These SNPs can then be utilized for phylogenetic analysis using a program such as MEGA version 5.04 [41].

### Visualization of genome reconstruction

The genome reconstruction provides an overview of the entire genome recovered for organisms identified from the metagenome sequences. The genome reconstruction of the identified organism is performed with the reference-based assembly [42]. The resulting contigs are merged as super scaffolds (http://abacas.sourceforge.net/Manual.html) and visualized with MAUVE [43].

## Results and Discussion

The goal of clinical metagenomics is often to identify the cause of infection amidst a veritable sea of host and microbial sequences. No two metagenomes are the same and broad variation exists due to the differences in microbial diversity and abundance as well as the size and architecture of genomes in the sampled community [44]. Other factors that dictate metagenome variation are the low amount of target DNA (often a pathogen), DNA from other microbes in the community, and the amount of host DNA, in addition to variation based on clinical sample type. The variation in metagenomes and the needs of researchers and clinicians makes it challenging to develop a “one-size-fits-all” method for analysis.

The characterization of community composition using microbial sequences can now be approached at three specific taxonomic levels: genus, species and strain/genotype (Figure S1), rather than the limited subfamily/genera that are the observable taxonomic units of 16S microbiome analysis. There are, however, fewer species- and strain-specific regions of the genome than genus-specific regions, given the relationships of genome composition with taxonomy. Increasing sequencing breadth across a genome allows for better taxonomic resolution of any organism present in a sample, especially for taxa that have been genetically well characterized. For metagenome data, single reads may map to multiple organisms either due to conserved microbial genomic regions (e.g., genus-specific genes) or due to the presence of closely related organisms in queried reference databases or the community being analyzed. Studies have shown metagenomic sequences share similar regions for even the simplest microbial communities [17], [45], [46]. Assigning each read to all mapped genomes might be an effective strategy as metagenome community analysis is unbiased and researchers may have no *a priori* knowledge about the community composition [38]. The genus specific reads will map to higher numbers of organisms followed by reads specific to species and sub-species/strains. The organism with the highest shared (genus-specific) regions, as well as unique regions, which generally belong to species- and strain-specific genes, will result in a higher percent of the genome mapped. The taxonomic rank and the detection resolution is proportional to sequencing throughput, richness of pathogen(s) in metagenome sampling and the availability of genomic data from the community members (e.g., target pathogens), or close relatives, in the reference database. We benchmarked the sensitivity and specificity of the detection step of the pipeline by evaluating simulated read libraries through identification of correct pathogen, corresponding percent read recalled, genome coverage detected, correct percentage of host reads filtered and false detection of host and/or non-host.

### Human Read Reduction

**Figure 2:**
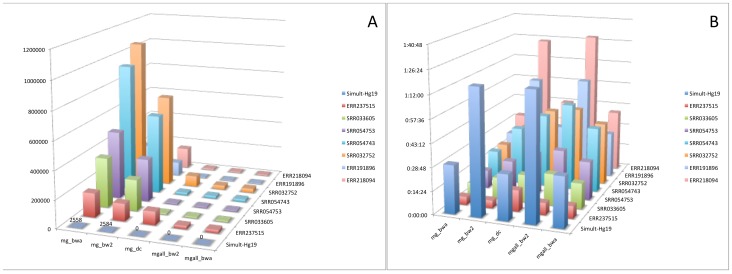
Benchmarking the human read reduction module of the pipeline. **A.** Total numbers of reads remaining after human read reduction with different filtration parameters **B.** Runtime for human read filtration with different aligner and filtration parameters (in minutes).

To detect the “needle” (e.g. pathogen reads), reducing the size of the “haystack” (non-target reads) is critical [6]. This starts with removing the overwhelming majority of reads, i.e. host DNA sequence. The efficiency of human read filtration can be measured by the total number of human reads removed from clinical samples. To test the effect on human read filtration with different parameters, seven whole genome sequencing datasets from humans and one simulated dataset created from human reference genome (Hg19) were analyzed. The mg_bwa and mg_bw2 uses only BWA and BOWTIE2 aligners only, while mgall_bw2 and mgall_bwa uses all five steps of pipeline including fast alignment with BOWTIE2 and BWA, respectively (Table 1). We found that use of a single aligner (mg_bwa/mg_bw2) is not always efficient in removing human reads. Utilizing all the features of the MetaGeniE pipeline (mgall_bw2 and mgall_bwa) allowed higher filtration of human reads (Figure 2-A). The runtime of single step (mg_bwa/mg_bw2) was faster than running all steps of human filtration (mgall_bwa/mgall_bw2) (Figure 2-B). Keeping all parameters the same, we found that the BWA aligner ran faster than BOWTIE2 (Figure 2-B) but that this increased speed comes at a cost; BOWTIE (mg_bw2) was more sensitive than BWA (mg_bwa) and correctly aligned a higher number of human reads (Figure 2-A). However, the total number of reads removed by mgall_bw2 and mgall_bwa (that utilizes all the steps of human filtration) was nearly equal, irrespective of whether the BWA or BOWTIE2 aligner was used.

Remaining human reads that were not filtered were aligned against the NCBI Refseq bacterial database. These “human” reads mapped to the bacterial database and as expected, were higher for single step alignment (*_mg_bw2) than with comprehensive human read reduction with pipeline (*_mgall_bw2) (Figure S2). The unfiltered human reads not only mapped incorrectly to microbial datasets, but also contributed to overall runtime during pathogen detection. Removal of human reads with high specificity is advantageous for sensitive clinical interpretation.

### Pathogen Detection

Metagenome datasets derived from clinical samples typically have analytical challenges such as a) the often extremely low abundance of pathogens; b) the analyzed sample may contain single infection (with only one dominant infection); c) contain multiple infectious agents from close relatives; and d) samples may house highly complex microbial communities (e.g. sputa from cystic fibrosis patients). The sequencing reads aligned against the reference genome(s) may have high divergence resulting from sequencing error or/and mutations. We created and analyzed simulated libraries based on varying community complexities to estimate the efficiency of pathogen detection.

#### Simple Community

Metagenome sequences are often processed as a single genome alignment to a reference genome [16]. In a single genome alignment, reads aligning to multiple loci in a reference genome are randomly assigned to a locus and SAMTools only parses these as “main” hits [47]. To evaluate the ability of MetaGeniE to distinguish a known target strain from its close relatives with our all-against-all strategy, we utilized *S. aureus* strain TCH1516 to assess the detection of a single infection by a known strain. *Staphylococcus* is well-characterized genus with high number of sequenced strains, allowing us to test the specificity of detecting the correct organism from not only among the many species of *Staphylococcus* in the reference genome database, but also from members of its own strain or subtype (i.e. ST8-MRSA-IVa/USA300). Typically the genus-specific regions of *Staphylococcus* are assigned to several, or all, of the members of the genus. Reads that contribute to unique regions, which may belong to its species (*S*. *aureus*), and strain-specific genes (clonal complex 5), will result in highest percent genome coverage of the correct organism. We were able to detect *S. aureus* TCH1516 in all the test sets as the top hit (highest genome reconstruction/coverage) even with lowest number of reads (i.e., 100 reads). This detection occurred even when single genome alignment was not able to report correct detection (Table S1). We found that the single alignment underestimates the genome coverage compared with the results from MetaGeniE, and the coverage detected by our approach approximated to the actual coverage detected (Table S2).

**Figure 3:**
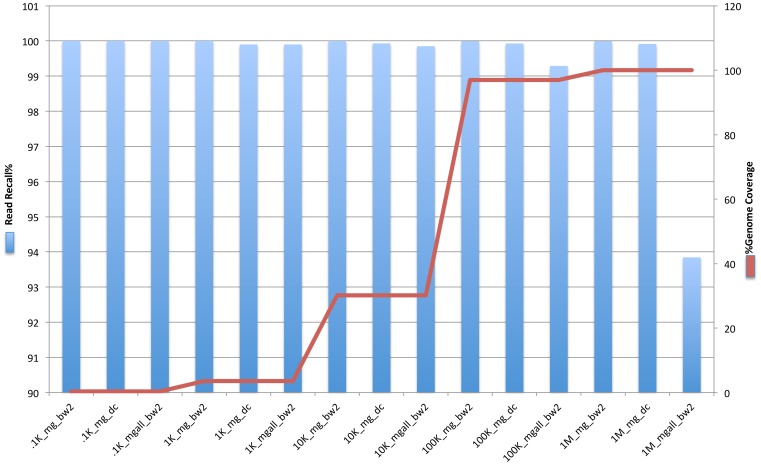
Effect of human filtration on percent genome coverage and read recall percentage of pathogen detection. The legends of the figure are prefixed with the number of reads (0.1K = 100; 1K = 1000; 10K = 10000; 100K = 100000; 1M = 1000000) followed by mg_bw2 for only fast alignment feature of human read reduction; mg_dc for all features of human read reduction except data compression; mgall_bw2 for all features of human read reduction module).

We also compared the effect on pathogen detection based on factors such as quantity of reads, percent genome coverage and read recall (reads aligning correctly to its genome of origin) percentage against different parameters available in human filtration module of the MetaGeniE (Figure 3). Read recall percentage is the percent of simulated reads that correctly align to the reference genome after human filtration. As the read number increased, the expected genome coverage percentage also increased; genome coverage reached 99.9% at 250K reads and thus had coverage across nearly the entire genome. The 250K reads were approximately the number of reads necessary to reconstruct the entire genome of *S. aureus* TCH1516 from the metagenome. As more reads were sequenced (simulated), a higher number of duplicate reads was also expected. Using the data compression feature of human filtration of pipeline (*_mgall_bw2) to remove duplicates reduced the read recall percentage but had no effect on genome coverage percentage or detection of the correct organism. The duplicate reads therefore did not add additional information; to manage computational scalability, removal of these duplicates improved MetaGeniE performance. We also found that using all the human filtration steps of the pipeline (*_mgall-bw2) as compared to using just fast alignment (*_mg_bw2) or not utilizing data compression (*_mg_dc) did not lead to underestimation of the percent genome coverage for correct pathogen detection.

#### Complex Community

The ability to detect and differentiate the members of the community in complex clinical samples, such as those from cystic fibrosis patients, should be helpful in generating insight for proper treatment. Shared regions are expected in even the simplest microbial communities so careful attention is necessary for these orthologs. The simulated library allowed us to evaluate the impact on detection due to the presence of multiple organisms in community with different genome sizes. We designed a simulated complex community of five bacteria based on a similar community composition that was previously detected from a cystic fibrosis clinical sample (See Methods). Simulated reads were generated from the reference genome of each of the five organisms and four libraries with different read numbers (i.e., 100, 1000, 10K, 100K per organism) were created. In metagenomes, many organisms may not have any complete or incomplete entries in the reference genome database. To test the specificity of detection of an unknown organism, *Veillonella dispar* ATCC 17748 was added to this complex community. This organism was not present in the bacterial reference genome database (RefSeq Build 60). Querying a large reference database usually results in detection of multiple organisms within same genus due to sequence homology. Therefore, for organism detection we selected the highest mapped genome percentage (i.e., the top hit) within the same genus. The correct detection was confirmed for all of the organisms except for *V. dispar* ATCC 17748 (Table S3). This indicates that the pipeline allowed detection of the correct organisms even in a complex community.

**Figure 4:**
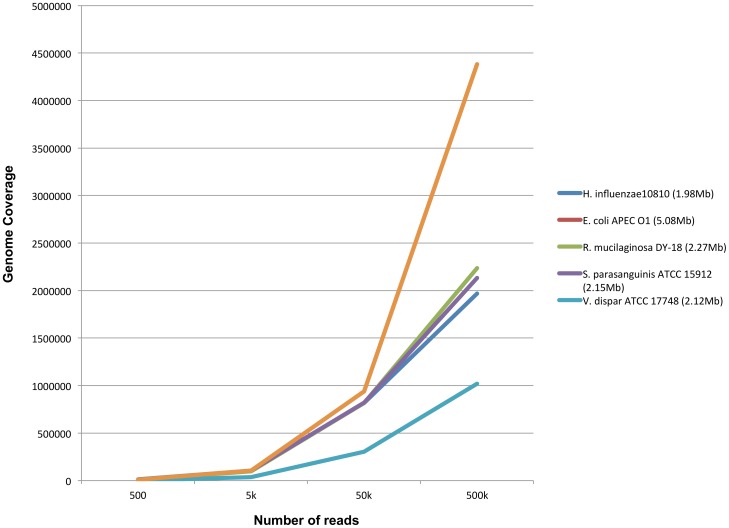
Detection of genomes in complex community. Relationship between genome size and genome coverage with increasing sequencing reads. Effect of detection on *E. coli* APEC O1 in simple and complex community.

Different genera in a complex community may share genomic regions. The robustness of detection can be measured by loss of sensitivity (i.e., genome coverage) of any organism in a complex versus simple community infection. We compared the percent genome coverage of *E. coli* APEC O1 as single pathogen and in complex community. We found no loss in percent genome coverage for the *E. coli* APEC O1 between simple and complex community and the trend for simple and complex community overlaps completely in the Figure 4. The all-against-all relationship between the reads and reference database, therefore, allows us to detect any organism without loss in sensitivity, which could potentially occur in samples containing organisms with shared genomic regions.

For simulated reads of *V. dispar* ATCC 17748 (not present in reference database), *V. parvula* DSM 2008 chromosome was detected as top hit with lower percent genome coverage compared to other hits (Figure 4). We can infer that true calls (i.e. detections) may not always be possible, given the limited, albeit growing, nature of genomic databases and the taxonomic resolution might decrease to genus, (e.g. *Veillonella* in this case).

### Co-infections

We were able to accurately detect and identify the target organism (as a top hit) for each taxon from multiple genera in a complex community as discussed above. However, some clinical samples will have pathogens from same species, for example co-infections with methicillin resistant *S. aureus* (MRSA) and methicillin sensitive *S. aureus* (MSSA). *Staphylococcus aureus* TCH1516 and *S. aureus* Newman belong to different clonal complexes (CC8 & CC5) and are abbreviated as MRSA and MSSA, respectively. To test the specificity of detecting and distinguishing these two distinct strains in clinical samples, we created co-infection libraries consisting of simulated reads from *S. aureus* Newman and *S*. *aureus* TCH1516 genomes.

**Figure 5:**
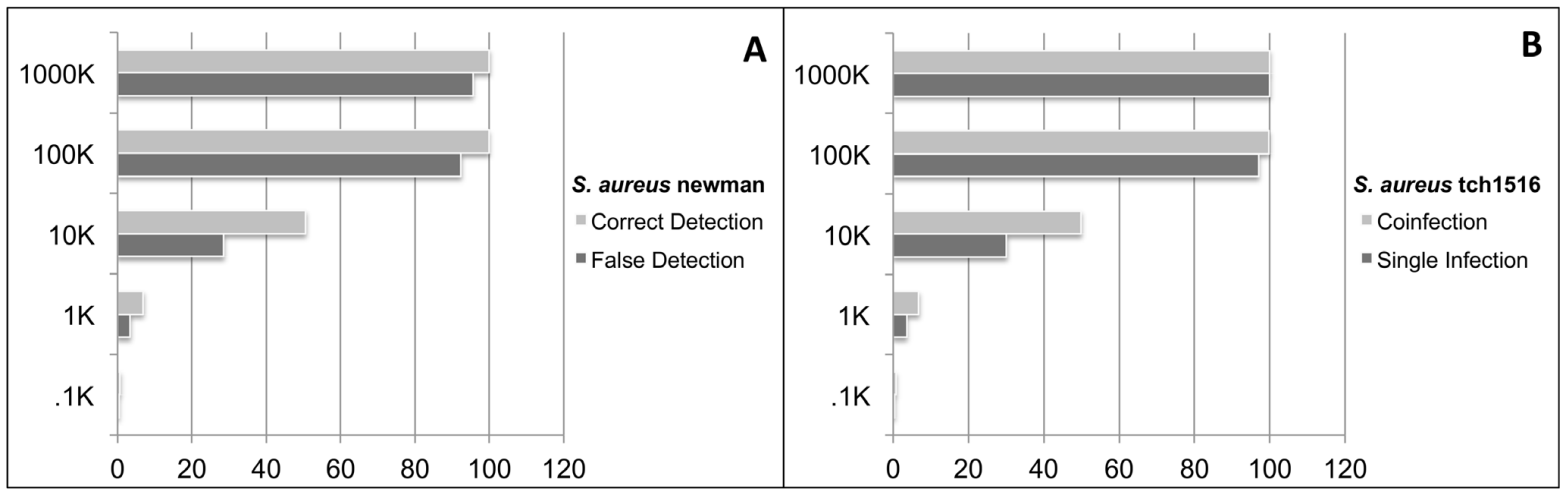
Comparison of detection of close relative in co-infection versus single infection. **A.** Comparison of percent genome coverage of true detection in co-infection versus false detection of *S. aureus* Newman. **B.** Comparison of percent genome coverage of *S. aureus* TCH1516 in co-infection versus simple infection.

The presence of *S. aureus* Newman in co-infection library (true positive) was compared with its detection in a single infection library (false positive) containing only simulated reads from the *S. aureus* TCH1516 genome (Figure 5-A). Any genome coverage percentage detected for *S. aureus* Newman in single infection library can be considered as false detection. The percent genome coverage of *S. aureus* Newman (false call) was slightly less than its true presence in multiple-infection library, due to contribution of homologous reads from *S. aureus* TCH1516. As summarized in (Table S4), *S. aureus* Newman ranked behind few other closely related genomes of *S. aureus* TCH1516 (CC5) in the single infection library (Table S5) but was detected as top hit in co-infection library (Table S6).

The co-infection library consisted of reads from both *S*. *aureus* TCH1516 and *S. aureus* Newman. Due to the all reads mapped against all reference strategy, the shared homology between these two organisms resulted in a higher percent genome coverage of *S. aureus* TCH1516 in the co-infection library than the single-infection library (Figure 5-B). The *S. aureus* TCH1516 was detected as top hit per genus in single infection library (Table S5) and as one of top hits with *S. aureus* Newman in co-infection library (Table S6). We can infer that although the “top hit per genus” detection was correct in identifying the correct strain in a co-infection, proper detection of the strains in a co-infection is difficult and will require additional validation.

### Diversity

Metagenome reads may have artificial variation due to sequencing error. The ability to assign these reads back to their genome can affect the sensitivity of detection. However, utilizing only a global aligner may result in loss of sensitivity of divergent reads detection. To incorporate these divergent reads for sensitive detection, we utilized BLAT, which is ∼500 times faster than preexisting tools with comparable sensitivity [35].

**Figure 6:**
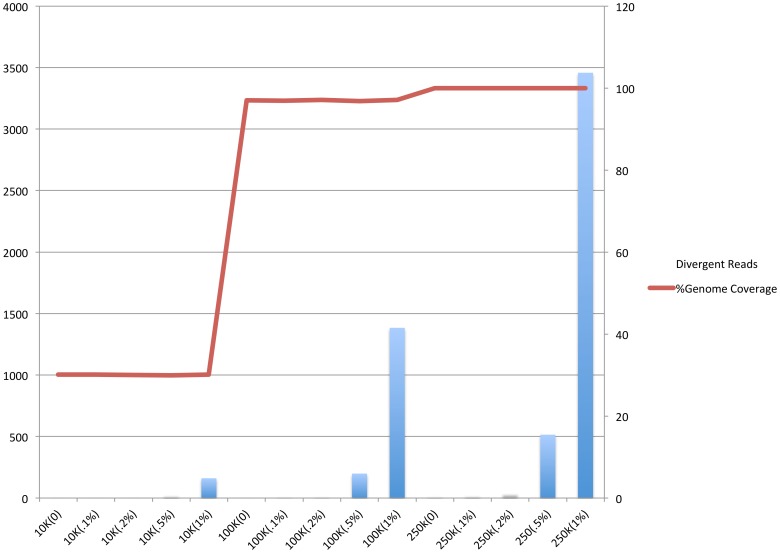
Relationship between percent genome coverage and read recall percentage with incremental divergence (i.e. error).

We designed the simulated reads from *S. aureus* TCH1516 genome with increasing amounts of error in the reads. To evaluate sensitivity to error, reads that the global aligner was unable to map, but were aligned by a local aligner (BLAT), were categorized as divergent reads. With increasing sequence divergence, higher numbers of reads were not aligned by global aligner (Figure 6). MetaGeniE is nonetheless able to incorporate these divergent reads through local alignment without a decrease in the genome coverage detected (Figure 6). In all 25 of the simulated test cases (0%, 0.1%, 0.2%, 0.5% and 1% divergence for 100, 1K, 10K, 100K, 250K reads), *S*. *aureus* TCH1516 was detected correctly in all except one: at 1% divergence with 100 reads. The limitation of detection for correct identification can therefore be seen at highest divergence with low number of reads.

### Clinical Samples

#### Workflow

Due to the variations and limitations in metagenome analyses and importance of detection accuracy given clinical perspective, the analyses of clinical samples might require a cycle of Detection → Validation → Confirmation (Figure S3). After detection of the pathogen likely responsible for the infection as well as assessing the rest of the microbial community, the validation of clinical datasets can be done through analysis such as SNP genotyping and BLAST analysis, depending on the number of reads aligned to the detected organism to more fully characterize the organism(s). These inferences from clinical datasets can finally be confirmed with laboratory test/culture, PCR, and/or patient's clinical history. We performed Detection→ Validation → Confirmation workflow to evaluate overall performance in the cystic fibrosis (CF) clinical dataset.

#### Detection

**Figure 7:**
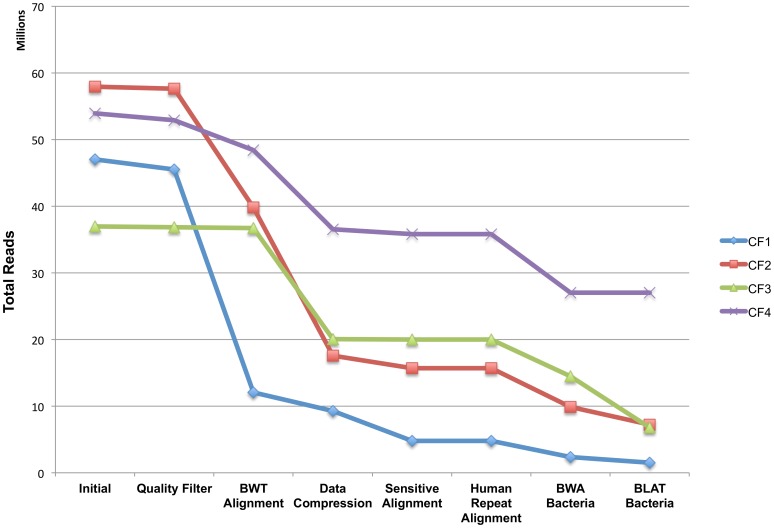
Sequential reduction of the metagenome reads for 4 clinical samples from cystic fibrosis patients. Data points represent the remaining reads after each processing step of the pipeline. First six data points (Initial, Quality Filter, BWT Alignment, Data Compression, Sensitive Alignment, Human Repeat Alignment) represent the Human Read Reduction and BWA Bacteria and BLAT Bacteria represent Pathogen Detection against bacterial database.

We first removed low quality, redundant and human reads with the MetaGeniE Read-Reduct module on the initial metagenomic reads (Figure 7). For the CF samples, the data were reduced 33–90%. The remaining reads after running the read filtration module were mapped against bacterial reference genome to detect pathogens. Different steps utilized by the pipeline have varying effects of reduction/filtration on these metagenomes (Figure 7). The total number of reads that mapped against the bacterial database was 24–68% for these four samples. The increase in number of reads mapping due to local alignment in these samples was 27–53% and therefore implementation of local alignment in the pipeline helped in aligning a higher number of divergent reads that increased the sensitivity for detection (Figure 7).

The mapped reads in the Patho-Detect module of the pipeline was utilized to understand the community with percent genome mapped for top-hit per genus for the CF samples (Table S7). The percent genome mapped (i.e., genomic reconstruction of the top hits for CF samples) ranged from 55–99% (Table S8). The four CF samples presented different genomic signatures (Table 2) and thus different communities in each patient (Table S8). Infectious agents in CF patients are acquired through nosocomial, social and environmental factors [48]–[50]. These pathogens, along with commensal microbiota, represent the microbial community in CF patients. The community for each CF metagenome sample was represented by normalized (%) genome coverage of top hit per each genus that was detected by MetaGeniE (Figure S4). Organisms from genera such as *Gemella*, *Granulicatella*, *Haemophilus*, *Neisseria* and *Streptococcus* are commonly found in the oral microbiome, including oral samples from CF patients [48], [49], [51], [52].

**Table 2:** Bacterial infection detected by MetaGeniE confirmed with the laboratory culture media.

Sample	Culture Report	Metagenome Detection
CF1	MRSA	*Staphylococcus aureus* subsp. *aureus* USA300 TCH1516
	ENCL	*Enterobacter cloacae* subsp. *cloacae* ATCC 13047
CF2	ECOL	*Escherichia coli* APEC O1
	HAEM	*Haemophilus influenzae* 10810
CF3	ECOL	*Escherichia coli* S88
	ENSP	*Enterococcus italicus* DSM 15952
CF4	MSSA	*Staphylococcus aureus* subsp. *aureus* str. Newman
	PSAR	*Pseudomonas* a*eruginosa* PAO1

**MRSA:** Methicillin resistant *Staphylococcus aureus*; **ENCL:** *Enterobacter cloacae*; **PSAR:** *Pseudomonas aeruginosa*; **MSSA:** Methicillin sensitive *S*. *aureus*; **ECOL:** *Escherichia coli*; **ENSP:** *Enterococcus* sp.; **HAEM:** *Haemophilus influenza*.

#### Validation

*SNP Genotyping*: Single nucleotide polymorphism (SNP) genotyping is widely used in analysis of WGS to accurately identify and discriminate between strains of a species [53]. Figure S5-1 represents the phylogenetic tree for the metagenome sequences mapping to top hit (*S. aureus* USA300 TCH1516) detected by pipeline for sample CF1. To confirm the accuracy of the detection of *S. aureus* USA300 TCH1516 for CF1 sample, the close relatives of available *S. aureus* from GenBank were downloaded and SNP genotyping was performed. We found that *S. aureus* USA300 TCH1516 detected by MetaGeniE is confirmed through SNP genotyping for CF1 and other CF samples (Figure S5). We are able to validate that detection at a high taxonomic level is possible in a clinical metagenome sample.

#### Genome Reconstruction and Visualization

We extracted and then assembled the reads mapped to these identified genomes to generate contigs and scaffolds. This pre-selection approach is different than assembling entire metagenome as this might result in chimeric contigs [17], [41], [42]. We were able to reconstruct all features of the identified MRSA and MSSA strains in CF1 and CF4 respectively (Figure S6). Due to low coverage, identified strains of sample CF2 and CF3 were not fully reconstructed.

#### Confirmation

The top hits for pathogen detection and community composition were confirmed in all four CF samples using culture-based methods from clinical laboratory (Table 2). The ability of MetaGeniE to correctly identify infections to the strain level, for example MRSA versus MSSA detection, demonstrates higher resolution than amplicon sequencing community analysis (e.g., 16S microbiome).

## Conclusions

Various features have been incorporated and validated in the MetaGeniE pipeline to improve computational scalability, speed, and accuracy, which allowed us to perform comprehensive analysis of the clinical samples from whole sample sequence data. We successfully tested the pipeline on various simulated clinical datasets, available public datasets and in-house sequenced clinical datasets.

## Supporting Information

Figure S1 **Hierarchical architecture of genomes and its relationship with sequencing throughput.**
(DOCX)

Figure S2 **Total numbers of reads aligning to bacterial database after human read filtration for human datasets.**
(DOCX)

Figure S3 **The workflow of the clinical sample analysis.**
(DOCX)

Figure S4 **Distribution of microbial community across the four cystic fibrosis samples.**
(DOCX)

Figure S5 **Phylogenetic tree representing the mapped reads from clinical dataset and the available genome in GenBank.** **S5-1.** Mapped reads from *Staphylococcus aureus* USA300 TCH1516. **S5-2.** Mapped reads from *Escherichia coli* APEC O1. **S5-3.** Mapped reads from *Staphylococcus aureus* Newman.
(DOCX)

Figure S6 **The visualization of the genomic reconstruction for the organism detected for four cystic fibrosis samples.**
(DOCX)

Table S1 **Comparison of genome coverage detection with single genome alignment versus metagenome alignment.**
(XLSX)

Table S2 **Comparison of single genome alignment and metagenome alignment with actual genome coverage.**
(DOCX)

Table S3 **Detection of organisms in complex community with metagenome alignment.**
(XLSX)

Table S4 **Rank of** ***Staphylococcus aureus*** **Newman and** ***Staphylococcus aureus*** **TCH1516 in single infection and co-infection library.**
(DOCX)

Table S5 **Detection of close relative in single infection library consisting of** ***Staphylococcus aureus*** **TCH1516.**
(XLSX)

Table S6 **Detection of close relative in co-infection library consisting of** ***Staphylococcus aureus*** **Newman and** ***Staphylococcus aureus*** **TCH1516.**
(XLSX)

Table S7 **Detection of organisms in four cystic fibrosis clinical samples with metagenome alignment.**
(XLSX)

Table S8 **Top five hits sorted by the genome coverage mapped per organism for four cystic fibrosis clinical samples.**
(DOCX)
