# Supplementary material for: MetaGeniE: Characterizing Human Clinical Samples Using Deep Metagenomic Sequencing
Source: PLoS One. 2014 Nov 3;9(11):e110915. doi: 10.1371/journal.pone.0110915 (PMC4218713; doi:10.1371/journal.pone.0110915)
Supplement: Figure S2 — Total numbers of reads aligning to bacterial database after human read filtration for human datasets. (DOCX) [file pone.0110915.s002.docx]

**MetaGeniE: Characterizing Human Clinical Samples Using Deep Metagenomic Sequencing**

Arun Rawat^1*^, David M. Engelthaler^1^, Elizabeth M. Driebe^1^, Paul Keim^1,2^, Jeffrey T. Foster^2,3*^

^1^Pathogen Genomics Division, Translational Genomics Research Institute, Flagstaff, Arizona, United States of America

^2^Center for Microbial Genetics and Genomics, Northern Arizona University, Flagstaff, Arizona, United States of America

^3^Department of Molecular, Cellular, and Biomedical Sciences, University of New Hampshire, Durham, New Hampshire, United States of America


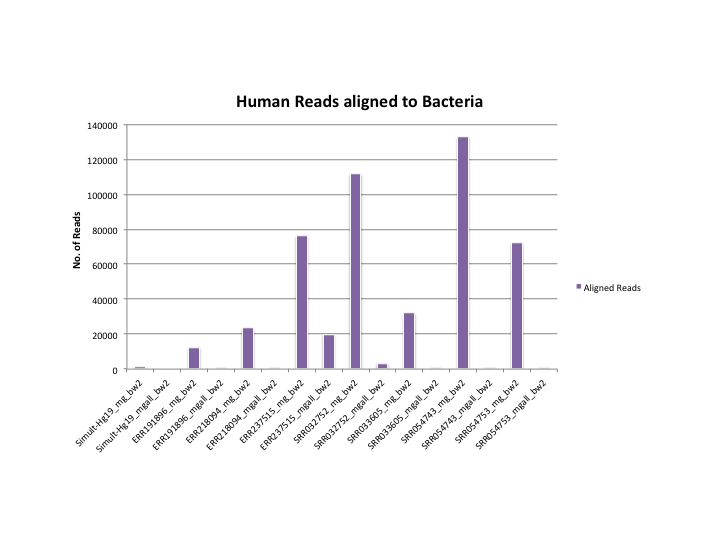


**Figure S2.** Total numbers of reads aligning to bacterial database after human read filtration for human datasets. The legends are prefixed with sample name (Simult-Hg19, ERR191896, ERR218094, ERR237515, SRR032752, SRR033605, SRR054743, SRR054753) followed by mg_bw2 and mgall_bw2 that represent utilizing single aligner and multiple feature of the pipeline respectively.
