# Supplementary material for: MetaGeniE: Characterizing Human Clinical Samples Using Deep Metagenomic Sequencing
Source: PLoS One. 2014 Nov 3;9(11):e110915. doi: 10.1371/journal.pone.0110915 (PMC4218713; doi:10.1371/journal.pone.0110915)
Supplement: Figure S5 — Phylogenetic tree representing the mapped reads from clinical dataset and the available genome in GenBank. S5-1. Mapped reads from Staphylococcus aureus USA300 TCH1516. S5-2. Mapped reads from Escherichia coli APEC O1. S5-3. Mapped reads from Staphylococcus aureus Newman. (DOCX) [file pone.0110915.s005.docx]

**MetaGeniE: Characterizing Human Clinical Samples Using Deep Metagenomic Sequencing**

Arun Rawat^1*^, David M. Engelthaler^1^, Elizabeth M. Driebe^1^, Paul Keim^1,2^, Jeffrey T. Foster^2,3*^

^1^Pathogen Genomics Division, Translational Genomics Research Institute, Flagstaff, Arizona, United States of America

^2^Center for Microbial Genetics and Genomics, Northern Arizona University, Flagstaff, Arizona, United States of America

^3^Department of Molecular, Cellular, and Biomedical Sciences, University of New Hampshire, Durham, New Hampshire, United States of America

***
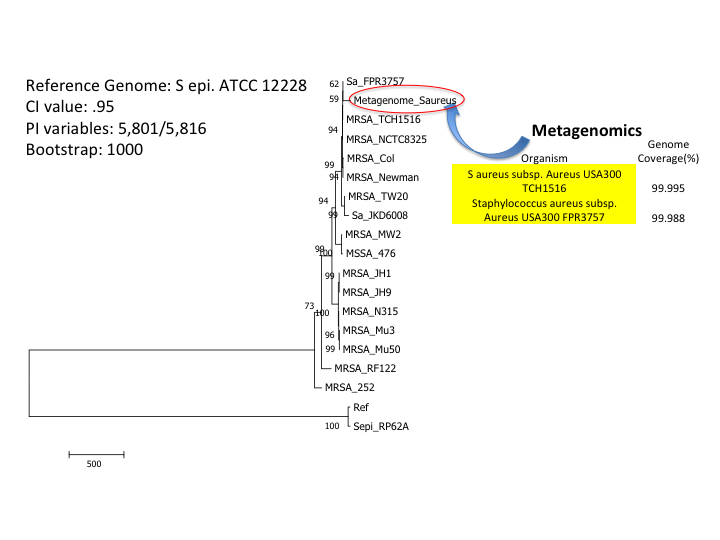
***

**Figure S5-1.** Phylogenetic tree representing the mapped reads of *Staphylococcus aureus* USA300 TCH1516 from clinical dataset and available genome in GenBank. (CI – Consistency Index; PI – Parsimony Informative).

**

**Figure S5-2.** Phylogenetic tree representing the mapped reads of *Escherichia coli* APEC O1 from clinical dataset and available genomes in GenBank.

**Figure S5-3.** Phylogenetic tree representing the mapped reads of *Staphylococcus aureus* Newman from clinical dataset and available genomes in GenBank. *Staphylococcus aureus* subsp. *aureus* str. Newman (MSSA) detected in CF4 sample that is confirmed by culture report is not accurately detected by SNP genotyping due to low depth of this organism in metagenome sample.
