# Supplementary material for: MetaGeniE: Characterizing Human Clinical Samples Using Deep Metagenomic Sequencing
Source: PLoS One. 2014 Nov 3;9(11):e110915. doi: 10.1371/journal.pone.0110915 (PMC4218713; doi:10.1371/journal.pone.0110915)
Supplement: Figure S6 — The visualization of the genomic reconstruction for the organism detected for four cystic fibrosis samples. (DOCX) [file pone.0110915.s006.docx]

**MetaGeniE: Characterizing Human Clinical Samples Using Deep Metagenomic Sequencing**

Arun Rawat^1*^, David M. Engelthaler^1^, Elizabeth M. Driebe^1^, Paul Keim^1,2^, Jeffrey T. Foster^2,3*^

^1^Pathogen Genomics Division, Translational Genomics Research Institute, Flagstaff, Arizona, United States of America

^2^Center for Microbial Genetics and Genomics, Northern Arizona University, Flagstaff, Arizona, United States of America

^3^Department of Molecular, Cellular, and Biomedical Sciences, University of New Hampshire, Durham, New Hampshire, United States of America

**Figure S6.** The visualization of the genomic reconstruction for the organism detected for four cystic fibrosis samples.
