# Supplementary material for: MetaGeniE: Characterizing Human Clinical Samples Using Deep Metagenomic Sequencing
Source: PLoS One. 2014 Nov 3;9(11):e110915. doi: 10.1371/journal.pone.0110915 (PMC4218713; doi:10.1371/journal.pone.0110915)
Supplement: Table S2 — Comparison of single genome alignment and metagenome alignment with actual genome coverage. (DOCX) [file pone.0110915.s008.docx]

**Table S2.** Comparison of accuracy of genome coverage detection in single genome alignment versus metagenome alignment with increasing read number

* Genome coverage of top hit reported even if the organism detected is incorrect.

The single alignment coverage is performed with BWA – SAMTools – BEDTools against multiple genome RefSeq dataset.

Metagenome alignment is performed with human read reduction and pathogen detection of pipeline against multiple genome RefSeq dataset.

The actual coverage is performed with BWA – SAMTools – BEDTools against single genome of *S.* *aureus* TCH1516 genome.

|  | **100** | **1K** | **10K** | **100K** | **250K** |
| --- | --- | --- | --- | --- | --- |
| **Single Alignment Coverage** | 0.014* | 0.059 | 0.62* | 5.116 | 11.307 |
| **MetaGenome Coverage** | 0.352 | 3.557 | 30.126 | 97.004 | 99.983 |
| **Actual Coverage** | 0.348 | 3.427 | 29.494 | 96.955 | 99.982 |

Actual coverage is slightly lower than the coverage detected by our pipeline as actual coverage is calculated from only BWA alignment while the pipeline calculate coverage from both BWA and BLAT alignment.
