# Supplementary material for: MetaGeniE: Characterizing Human Clinical Samples Using Deep Metagenomic Sequencing
Source: PLoS One. 2014 Nov 3;9(11):e110915. doi: 10.1371/journal.pone.0110915 (PMC4218713; doi:10.1371/journal.pone.0110915)
Supplement: Table S4 — Rank of Staphylococcus aureus Newman and Staphylococcus aureus TCH1516 in single infection and co-infection library. (DOCX) [file pone.0110915.s010.docx]

**Table S4.** Rank of *S.* *aureus* Newman and *S.* *aureus* TCH1516 in single infection and co-infection library. FP: False Positive; TP: True Positive

|  | **Ranking of *Sa. Newman*** | | **Ranking of *Sa. TCH1516*** | |
| --- | --- | --- | --- | --- |
| **Reads** | **FP** | **TP** | **Single Inf (TP)** | **Co-Inf (TP)** |
| **0.1K** | 5 | 1 | 1 | 2 |
| **1K** | 4 | 1 | 1 | 3 |
| **10K** | 5 | 1 | 1 | 3 |
| **100K** | 5 | 1 | 1 | 2 |
