# Supplementary material for: MetaGeniE: Characterizing Human Clinical Samples Using Deep Metagenomic Sequencing
Source: PLoS One. 2014 Nov 3;9(11):e110915. doi: 10.1371/journal.pone.0110915 (PMC4218713; doi:10.1371/journal.pone.0110915)
Supplement: Table S8 — Top five hits sorted by the genome coverage mapped per organism for four cystic fibrosis clinical samples. (DOCX) [file pone.0110915.s014.docx]

**Table S8.** Top five hits sorted by the genome coverage mapped per organism for four cystic fibrosis samples.

| **Sample** | **Organism** | **Genome Coverage %** | **Genome Coverage** | **Genome Size** | |
| --- | --- | --- | --- | --- | --- |
| **CF1** | *Staphylococcus aureus* subsp. *aureus* USA300 TCH1516 | 99.995 | 2872779 | 2872915 |  |
|  | *Streptococcus sanguinis* SK36 | 75.607 | 1805813 | 2388435 |  |
|  | *Enterococcus faecalis* V583 | 50.015 | 1609507 | 3218031 |  |
|  | *Rothia mucilaginosa* DY-18 | 42.98 | 973323 | 2264603 |  |
|  | *Granulicatella adiacens* ATCC 49175 genomic scaffold | 88.068 | 669773 | 760519 |  |
| **CF2** | *Escherichia coli* APEC O1 | 93.321 | 4742614 | 5082025 |  |
|  | *Shigella sonnei* 53G | 73.695 | 3676268 | 4988504 |  |
|  | *Streptococcus parasanguinis* ATCC 903 | 82.959 | 1744143 | 2102412 |  |
|  | *Haemophilus influenzae* 10810 | 78.98 | 1565017 | 1981535 |  |
|  | *Veillonella dispar* ATCC 17748 genomic scaffold | 61.605 | 958312 | 1555587 |  |
| **CF3** | *Enterobacter cloacae* subsp. *cloacae* ATCC 13047 | 55.936 | 2972741 | 5314581 |  |
|  | *Klebsiella oxytoca* KCTC 1686 | 35.327 | 2110459 | 5974109 |  |
|  | *Streptococcus salivarius* CCHSS3 | 93.861 | 2081061 | 2217184 |  |
|  | *Veillonella dispar* ATCC 17748 | 85.802 | 1334727 | 1555587 |  |
|  | *Escherichia coli* S88 | 20.267 | 1019880 | 5032268 |  |
| **CF4** | *Staphylococcus aureus* subsp. *aureus* str. Newman | 73.3 | 2110219 | 2878897 |  |
|  | *Streptococcus salivarius* CCHSS3 | 92.536 | 2051701 | 2217184 |  |
|  | *Prevotella pallens* ATCC 700821 | 84.552 | 1957298 | 2314907 |  |
|  | *Fusobacterium nucleatum* subsp. *polymorphum* ATCC 10953 | 71.999 | 1749354 | 2429698 |  |
|  | *Haemophilus parainfluenzae* T3T1 | 74.169 | 1547812 | 2086875 |  |
